# Association between beta-2 adrenergic receptor variants and clinical outcomes in children and adolescents with acute asthma

**DOI:** 10.31744/einstein_journal/2022AO6412

**Published:** 2022-03-17

**Authors:** Fábio Pereira Muchão, Andréa Vieira de Souza, Juliana Miguita e Souza, Luiz Vicente Ribeiro Ferreira da Silva

**Affiliations:** 1 Hospital das Clínicas Faculdade de Medicina Universidade de São Paulo São Paulo SP Brazil Instituto da Criança, Hospital das Clínicas, Faculdade de Medicina, Universidade de São Paulo, São Paulo, SP, Brazil.; 2 Instituto Israelita de Ensino e Pesquisa Albert Einstein Hospital Israelita Albert Einstein São Paulo SP Brazil Instituto Israelita de Ensino e Pesquisa Albert Einstein, Hospital Israelita Albert Einstein, São Paulo, SP, Brazil.

**Keywords:** Asthma, Polymorphism, genetic, Receptors, adrenergic, beta-2, Bronchodilator agents, Metered dose inhalers, Nebulizers and vaporizers, Hospitalization

## Abstract

**Objective:**

To investigate whether different genotypes of p.Arg16Gly, p.Gln27Glu, p.Arg19Cys and p.Thr164Ile variants interfere in response to treatment in children and adolescents with moderate to severe acute asthma.

**Methods:**

This sample comprised patients aged 2 to 17 years with a history of at least two wheezing episodes and current moderate to severe asthma exacerbation. All patients received multiple doses of albuterol and ipratropium bromide delivered via pressurized metered-dose inhaler with holding chamber and systemic corticosteroids. Hospital admission was defined as the primary outcome. Secondary outcomes were changes in forced expiratory volume in the first second after 1 hour of treatment, and for outpatients, length of stay in the emergency room. Variants were genotyped by sequencing.

**Results:**

A total of 60 patients were evaluated. Hospital admission rates were significantly higher in carriers of the genotype AA relative to those with genotype AG or GG, within the p.Arg16Gly variant (p=0.03, test χ^2^, alpha=0.05). Secondary outcomes did not differ between genotypes.

**Conclusion:**

Hospital admission rates were significantly higher among carriers of the genotype AA within the p.Arg16Gly variant. Trial registration: ClinicalTrials.gov: NCT01323010

## INTRODUCTION

Asthma is a highly prevalent condition in childhood. Asthma exacerbation is a common cause of emergency visit and, hospital admission, school absenteeism and loss of quality of life in children.^(1-3)^Asthma attacks may also have negative impacts on asthma-related lung function. Maintenance treatment aimed to prevent exacerbations and appropriate management of these attacks are vital to minimize lung function compromise.^(4)^

Asthma is a heterogeneous condition. Wide variations regarding age of onset, presence or absence of atopy, severity and reversibility of airway obstruction, among other factors, have been reported. Hence, associations between phenotype and endotype-related factors and different clinical outcomes, including the occurrence and severity of exacerbations, have been increasingly reported.^(1-3)^

Short-acting bronchodilators are the cornerstone of asthma attack treatment. In mild attacks, a few doses given at home are enough to relieve symptoms. However, moderate to severe attacks require multiple doses at short intervals, especially in the first hour of treatment (usually, every 20 minutes,).^(1-3,5,6)^ The efficacy and safety of these drugs - particularly albuterol- when delivered via pressurized metered-dose inhalers with holding chambers (with mask or mouthpiece) have been demonstrated in several scientific studies.^(1-3,5,6)^ Therefore, the role of beta-2 adrenergic receptors (the target of bronchodilators) and the respective codifying gene (*ADRB2*) must be understood.^(7,8)^

The beta-2 adrenergic receptor is a member of a family of seven transmembrane receptors. This receptor contains 413 amino acids, with a molecular weight of approximately 46.500 daltons. Type 1 and type 3 beta adrenergic receptors are found in the cardiac muscle and fat tissue, respectively, whereas type 2 receptors are found primarily in airway smooth muscles and other lung cells, such as mast cells, epithelial cells, endothelial cells and type II pneumocytes. These receptors tend to be more abundant the higher the airway generation, and are present in particularly high numbers in small airways.^(9)^

*ADRB2* has 1,242 base pairs and is located in the long arm of chromosome 5 (5q 31-32). More than 80 variants of the *ADRB2* gene have been described.^(7,8,10-12)^Single nucleotide variants such as c.46A>G, p.Arg16Gly (rs1042713), c.79C>G, p.Gln27Glu (rs1042714) and c.-47C-T, p.Arg19Cys (rs1042711) may interfere with the downregulation of beta-2 receptor expression. In turn, the c.491C>T, p.Thr164Ile (rs1800888) variant is associated with reduced binding affinity for bronchodilators.^(13-15)^ Evidence suggested all four variants interfere with responses to bronchodilators in terms of lung function and other clinical outcomes, such as hospital admission and length of stay at intensive care unit (ICU), among others.^(7,8,11,16-18)^

## OBJECTIVE

To investigate whether specific genotypes of the p.Arg16Gly, p.Gln27Glu, p.Arg19Cys and p.Thr164Ile variants interfere in response to treatment in children and adolescents with moderate to severe acute asthma.

## METHODS

Variants c.46A>G, p.Arg16Gly (rs1042713); c.79C>G, p.Gln27Glu (rs1042714); c.491C>T, p.Thr164Ile (rs1800888) and c.-47C-T, p.Arg19Cys (rs1042711) were investigated.^(19)^

Data of patients in this sample were extracted from a prior study investigating the efficacy and safety of increasing doses of albuterol in children with acute asthma.^(5)^ Patients were recruited between January 2012 and January 2014, at the following locations: *Serviço de Consultas de Urgência e Triagem* of the *Instituto da Criança do Hospital das Clínicas, Faculdade de Medicina* of *Universidade de São Paulo* (USP), *Hospital Municipal Dr. Moysés Deutsch* (M’Boi Mirim) and *AMA Vila Prel Antônio Bernardes de Oliveira*, in São Paulo (SP). The investigation of *ADRB2* gene variants was conducted at *Instituto Israelita de Ensino e Pesquisa Albert Einstein* of *Hospital Israelita Albert Einstein*, in São Paulo. Data analysis was carried out at *Unidade de Pneumologia* of *Instituto da Criança* of *Hospital das Clínicas, Faculdade de Medicina,* USP.

Patients aged 2 to 17 years with a history of at least two wheezing episodes and current moderate to severe exacerbation according to the Pediatric Respiratory Assessment Measure (PRAM score ≥5) were included.^(20)^ Exclusion criteria were as follows: preexisting chronic disease, initial clinical presentation requiring immediate ventilatory support or subcutaneous or intravenous administration of bronchodilators in the first hour of treatment, decreased level of consciousness and use of beta-agonists 4 hours or systemic corticosteroids 24 hours prior to arrival.

Patients received multiple doses of albuterol and ipratropium bromide delivered via pressurized metered-dose inhalers with holding chambers. A minimum of three doses of albuterol and ipratropium were given within the first hour, followed by subsequent doses as needed. Nominal doses per treatment varied according to body weight and group (study or control) to which patients were allocated in the original study that gave rise to this trial (data published elsewhere).^(5)^ Patients received systemic corticosteroids (2mg/kg of prednisolone or methylprednisolone; maximum dose, 60mg) and were reassessed within 1 hour, then every 30 minutes. Patients who failed to meet hospital discharge criteria (PRAM ≤3 and pulse oximetry ≥92%)^(5)^ were hospitalized.

Need of hospital admission was defined as the primary outcome. Secondary outcomes were changes in forced expiratory volume in the first second (FEV_1_, measured at baseline and after 1 hour of treatment) and length of stay in the emergency room (ER) (in the case of outpatients).

Guardians and subjects aged over 12 years signed an informed consent term. This protocol was approved by the Ethics Committee of institutions involved. This study was registered on the Clinical Trials platform (NCT01323010) https://clinicaltrials.gov/ct2/show/NCT01323010 and conducted in compliance with Helsinki Declaration principles.

The region of the *ADRB2* gene encoding the p.Arg16Gly, p.Gln27Glu, p.Arg19Cys and p.Thr164Ile variants was amplified using polymerase chain reaction (PCR) and the following starting oligonucleotides: 5’ CTG AGT GTG CAG GAC GAG TC 3’, 5’ AAG TAG TTG GTG ACC GTC TGC AG 3’, 5’ CTG CAG ACG GTC ACC AAC TAC T 3’ and 5’ GAA GAG GCA ATG GCA TAG GCT 3’.^(19)^ Sample DNA was extracted using Gentra^®^ Puregene^®^ DNA Blood Kit (Qiagen, Hilden, Germany). The PCR for both fragments was prepared using approximately 200ng of sample DNA, 5µM of primers and 2.5U of Platinum™ Taq Polymerase in Taq DNA Polymerase 1x buffer (Invitrogen/Life Technologies, Thermo Fisher Scientific, Waltham, MA, United States) to obtain a final reaction volume of 50µL. The following amplification conditions were used in both cases: 95^o^C for 3 minutes followed by 35 cycles at 94^o^C for 40 seconds, 65^o^C for 40 seconds and 72^o^C for 40 seconds, plus a final extension step at 72^o^C for 5 minutes in a thermocycler (Mastercycler^®^, Eppendorf, Hamburg, Germany). Resultant amplimeres (361 and 422 base pairs respectively) were purified by column sequencing using PureLink^®^ PCR Purification Kit (Invitrogen/Life Technologies, Thermo Fisher Scientific, Waltham, MA, United States).

Purified samples were prepared for sequencing reactions using the BigDye™ Terminator Cycle Sequencing v.3.1 Kit (Applied Biosystems/Life Technologies, Thermo Fisher Scientific, Waltham, MA, United States): approximately 50ng of PCR product were added to the mixture containing 1µL of BigDye™ Terminator v.3.1- RR-100 in 1.5µL of reaction buffer (5x) and 3.2pmol/µL of primer for 10µL of final reaction volume. The sequencing reaction was run under the following conditions: 96^o^C for 1 minute followed by 30 cycles at 96^o^C for 10 seconds, 50^o^C for 5 seconds and 60^o^C for 4 minutes in a thermocycler (ABI 9600, Applied Biosystems/Life Technologies, Thermo Fisher Scientific, Waltham, MA, United States). Samples were then purified according to the ethanol/ethylenediamine tetraacetic acid (EDTA) fragment precipitation protocol for 10µL reaction, as per Kit user instructions.

The final product was eluted in 10µL of Hi-Di™ formamide (Applied Biosystems/Life Technologies, Thermo Fisher Scientific, Waltham, MA, United States). Immediately prior to capillary electrophoresis in ABI 3500xl sequencer (Applied Biosystems/Life Technologies, Thermo Fisher Scientific, Waltham, MA, United States), strands were denaturated at 95^o^C for 5 minutes, followed by material incubation at 40^o^C.

Sequencing results were analyzed using software (Chromas Pro v.3.1). Sequences were submitted to the Basic Local Alignment Search Tool (BLAST) platform of the National Center for Biotechnology Information (NCBI), available at http://blast.ncbi.nlm.nih.gov/.

### Statistical analysis

Qualitative variables were analyzed using the test χ^2^ or the Fisher’s exact test. The adherence test χ^2^ was used to compare the frequency of selected alleles in the study and the general population. Normally and asymmetrically distributed quantitative variables were analyzed using the *t* test for mean comparisons and the Kruskal-Wallis test, respectively. Repeated measures analysis of variance (ANOVA) was used whenever there were multiple measures over time. The level of significance was set at 5% (alpha=0.05). Statistical analyses were performed using SPSS, version 20.0 (IBM, Amonk, NY, United States).

In the protocol that gave rise to this study, a sample size of 124 patients was initially estimated to detect a difference of 30 minutes in length of stay in the emergency room between patients in the study and the Control Group, with a statistical power of 80% (data published elsewhere).^(5)^

## RESULTS

Genotyping was carried out in 60 out of 119 patients in this sample. In remaining patients, extraction attempts failed to yield viable DNA for *ADRB2* gene sequencing. Demographic characteristics of patients included in this study are shown in table 1.

**Table 1:** Demographic characteristics of patients included

Characteristic	
Age, years	7.22±3.68
Weight, kg	24.09±14.75
Ethnicity	
White	17 (28.3)
Black	5 (8.3)
Mixed	30 (50)
Not informed	8 (13.3)
Sex	
Male	31 (51.7)
Female	29 (48.3)

Results expressed as mean±standard deviation or n (%).

Patients were aged 2.02 to 15.18 years (minimum and maximum age, respectively). Mixed patients prevailed in this sample. Sex distribution was similar. *ADRB2* gene sequencing results, frequency of alleles and comparisons with the general population are shown in table 2.

**Table 2:** Genotypic and allelic frequency for p.Arg16Gly, p.Gln27Glu, p.Arg19Cys and p.Thr164Ile variants in patients with acute asthma

Variant	Genotype n (%)			Allele frequency in the study	Allele frequency in the general population^(21)^
p.Arg16Gly	AA: 23 (38.33)	AG: 16 (26.67)	GG: 21 (35)	A: 0.52 G: 0.48	A: 0.39 G: 0.61
p.Gln27Glu	CC: 53 (88.33)	CG: 4 (6.67)	GG: 3 (5)	C: 0.92 G: 0.08	C: 0.60 G: 0.40
p.Arg19Cys	TT: 51 (85)	TC: 6 (10)	CC: 3 (5)	T: 0.90 C:0.10	T: 0.61 C: 0.39
p.Thr164Ile	CC: 57 (95)	CT: 3 (5)	TT: 0 (0)	C: 0.97 T: 0.03	C: 0.99 T: 0.01

Source: U.S. National Library of Medicine. National Center for Biotechnology Information (NCBI). dbSNP. Bethesda: NCBI; s.d. [cited 2020 Dec 15]. Available from: https://www.ncbi.nlm.nih.gov/snp/^(21)^

The distribution of variant p.Arg16Gly was relatively uniform among three genotypes. In contrast, genotypes CC, TT and CC were clearly predominant in variants p.Gln27Glu, p.Arg19Cys and p.Thr164Ile, respectively. The frequency of variant p.Arg16Gly, p.Gln27Glu and p.Arg19Cys alleles differed between the study and the general population (p<0.05 in all three cases), whereas the frequency of variant p.Thr164Ile alleles was similar (p=0.09). This may have been due to the small sample size or the fact that this study was based on a selected sample (patients aged 2 to 17 years with moderate to severe acute asthma) with high levels of miscegenation, among other reasons.

No other variants were found.

Ethnic characterization of patients according to different genotypes of each variant is shown in table 3.

**Table 3:** Ethnic characterization of patients according to different genotypes

Variant and genotype	White	Black	Mixed	Not informed	p value comparison between ethnicities and genotypes*
p.Arg16Gly					p=0.168
AA	6	3	13	1	
AG	8	0	6	2	
GG	3	2	11	5	
p.Gln27Glu					p=0.549
CC	14	5	28	6	
CG	2	0	2	0	
GG	1	0	0	2	
p.Arg19Cys					p=0.284
TT	13	5	27	6	
TC	2	0	3	1	
CC	2	0	0	1	
p.Thr164Ile					p=0.814
CC	16	5	29	7	
CT	1	0	1	1	
TT	0	0	0	0	

* χ^2^ test.

For the four variants investigated in this study, the majority of patients were mixed. Ethnicity was not associated with different genotypes of the same variant.

Increase in FEV_1_ after 1 hour and ER length of stay did not differ according to genotype for ther different variants studied (comparison of different genotypes within variants).

However, hospital admission rates differed significantly (p=0.03; χ^2^ test) between genotypes whitin the p.Arg16Gly variant. Of nine hospitalized patients, seven were homozygous for the allele corresponding to arginine in position 16 (genotype AA), one was heterozygous (genotype AG) and one was homozygous for the allele corresponding to glycine (genotype GG). Hospital admission rates did not differ between genotypes for the other variants.

## DISCUSSION

In this study, the AA genotype (p.Arg16Gly) was clearly associated with higher hospitalization rates. In spite of almost equal distribution across the three genotypes, seven out of nine hospitalized patients were homozygous for the arginine allele. Different findings have been reported by Martinez et al., in a large longitudinal asthma study investigating responses to a single dose of albuterol in children. In that study, the odds of positive response to the bronchodilator, in the case of p.Arg16Gly variant, were 5.3-fold higher in patients with the AA genotype, and 2.3-fold higher in heterozygous individuals, relative to those with the GG genotype.^(11)^

Studies investigating associations between *ADRB2* gene variants and response to bronchodilators or disease severity in patients suffering from asthma and chronic obstructive pulmonary disease yielded conflicting results in different populations.^(7,8,22-24)^ In a national study conducted by de Paiva et al., in the matter of the p.Arg16Gly variant, who were homozygous for the arginine allele had a higher risk of asthma development. However, this variant alone was not associated with asthma severity.^(25)^ In contrast, in an investigation carried out by Scichilone et al., with 84 asthma patients, the frequency of genotype AA of this same variant was significantly higher in patients with severe relative to patients with mild or moderate asthma (55% and 22%, respectively).^(26)^

In other studies, genotype AA (p.Arg16Gly) was associated with poor chronic obstructive pulmonary disease control and greater need of antimicrobial treatment due to exacerbations.^(10)^ Genotype AA (p.Arg16Gly) may also be associated with higher risk of acute attacks, particular in regular users of long-acting bronchodilators.^(27,28)^ Along the same lines, Turner et al. conducted a meta-analysis of data from five cross-sectional studies reporting on *ADRB2* genotyping, type of treatment and exacerbations in 4,226 children. In that analysis, the odds ratio of exacerbation was 1.52 (95% confidence interval – 95%CI – 1.17-1.99; p=0.0021) for each copy of the allele A, in 624 children treated with inhaled corticosteroids and long-acting bronchodilators in the case of the variant p.Arg16Gly.^(29)^ Finally, in a similar investigation of patients with acute asthma, Carrol et al. reported significantly longer in-hospital stay (including ICU) in patients with severe asthma who were genotype AA or AG carriers relative to genotype GG carriers (p.Arg16Gly).^(18)^

Findings of this study are in keeping with those of prior investigations associating the genotype AA (p.Arg16Gly) with greater asthma severity or risk of exacerbation. Allele A (p.Arg16Gly) may also be related to poorer response to bronchodilators in patients exposed to multiple doses of such drugs, as observed in this study.^(18)^ Given only patients with moderate to severe acute asthma treated with multiple doses of albuterol were included, higher hospital admission rates among genotype AA carriers (p.Arg16Gly) in this sample is expected.

Different allele frequencies between the study and the general population do not invalidate findings presented, since outcomes were compared between genotypes within each variant.

Conflicting results regarding the effect of *ADRB2* variants across different studies may be related to several factors, such as different therapeutic approaches to asthma at different locations, and use of different criteria for asthma diagnosis and allocation of subjects to referral services, which lead to non-random allocation of patients with different degrees of asthma severity, among others.^(25)^

The frequency of *ADRB2* variants varies according to the ethnic composition of different populations. Given the high degree of miscegenation in this sample, more studies are warranted not only to better characterize the frequency of these different variants, but also to investigate association with different factors, such as asthma severity, response to bronchodilators and higher risk of unfavorable clinical outcomes in cases of acute asthma (hospital or ICU admission, need of mechanical ventilation, etc.). Tools capable of providing a deeper understanding of phenotypic and genotypic aspects of asthma patients will certainly contribute to personalized treatment and optimization of strategies aimed to prevent unfavorable outcomes. Short-acting bronchodilators are not the only therapeutic alternative for management of acute asthma. Should identification of patients with poor response to bronchodilators be possible, other effective adjuvant medications could be more often indicated. The following examples can be given: anticholinergic agents (*i.e*., inhaled ipratropium bromide), early administration of systemic corticosteroids and intravenous administration magnesium sulfate in more severe cases.^(1-3,30-32)^ Other alternatives, such as use of intravenous bronchodilators, theophylline and helium-oxygen mixture, are controversial. In contrast, use of inhaled magnesium sulfate is being increasingly reported, with a good safety profile.^(33-35)^

Finally, regardless of genetic profile, the significance of appropriate therapeutic maintenance of asthma patients, wich basis is the use of inhaled corticosteroids alone or combined with long-acting bronchodilators, must be emphasized. Excessive, isolated use of bronchodilators is a well-established risk factor for asthma exacerbation.^(1)^

Some limitations of this study must be acknowledged, especially regarding sample size. Larger samples would certainly enable better characterization of the prevalence of different genotypes of *ADRB2* variants in pediatric patients with acute asthma. A large proportion of patients in this sample was aged less than 6 years. Therefore, spirometry could only be performed in 23 children. This may have interfered with the investigation of potential effects of different genotypes on spirometric parameters. As previously alluded to, constraints related to DNA extraction from blood samples precluded *ADRB2* genotyping in a large number of patients, with negative impacts on sample size. Lack of data on prior maintenance treatment must also be emphasized.

## CONCLUSION

Hospital admission rates were significantly higher in carriers of the genotype AA relative to those with genotypes AG or GG (within the p.Arg16Gly variant). Hospital admission rates did not differ between genotypes for the remaining variants.

Changes in forced expiratory volume in the first second and length of stay in the emergency room did not differ between genotypes of variants investigated. Further studies are warranted for better characterization of the frequency of different genotypes, and the impact of beta 2 adrenergic receptor gene variants on therapeutic management of acute asthma in Brazilian children and adolescents.

## References

[1] . Global Innicaitive for Asthma. Global Strategy for Asthma Management and Prevention (2020 update). Fontana (WI): GINA; 2020 [cited 2020 Dez 12]. Available from: https://ginasthma.org/wp-content/uploads/2020/06/GINA-2020-report_20_06_04-1-wms.pdf

[2] . Pizzichini MM, Carvalho-Pinto RM, Cançado JE, Rubin AS, Cerci Neto A, Cardoso AP, et al. 2020 Brazilian Thoracic Association recommendations for the management of asthma. J Bras Pneumol. 2020;46(1):e20190307. 10.1590/1806-3713/e20190307 PMC7462684 32130345

[3] . Healthcare Improvement Scotland. Scottish Intercollegiate Guidelines Network (SIGN). SIGN 158. British guideline on the management of asthma. Edinburgh: SIGN; 2019 [cited 2020 Dec 11]. Available from: https://www.brit-thoracic.org.uk/document-library/guidelines/asthma/btssign-guideline-for-the-management-of-asthma-2019/

[4] . Pijnenburg MW, Fleming L. Advances in understanding and reducing the burden of severe asthma in children. Lancet Respir Med. 2020;8(10):1032-44. Review. 10.1016/S2213-2600(20)30399-4 32910897

[5] . Muchão FP, Souza JM, Torres HC, De Lalibera IB, de Souza AV, Rodrigues JC, et al. Albuterol via metered-dose inhaler in children: lower doses are effective, and higher doses are safe. Pediatr Pulmonol. 2016;51(11):1122-30. 10.1002/ppul.23469 27171324

[6] . Benito Fernández J, Trebolazabala Quirante N, Landa Guarriz M, Mintegi Raso S, González Díaz C. [Bronchodilators via metered-dose inhaler with spacer in the pediatric emergency department: what is the dosage?] An Pediatr (Barc). 2006;64(1):46-51. Spanish. 10.1016/s1695-4033(06)70008-x 16539916

[7] . Hizawa N. Pharmacogenetics of β2-agonists. Allergol Int. 2011;60(3):239-46. Review. 10.2332/allergolint.11-RAI-0317 21681016

[8] . Hizawa N. Beta-2 adrenergic receptor genetic polymorphisms and asthma. J Clin Pharm Ther. 2009;34(6):631-43. Review. 10.1111/j.1365-2710.2009.01066.x 20175796

[9] . Johnson M. The beta-adrenoceptor. Am J Respir Crit Care Med. 1998;158(5 Pt 3):S146-53. Review. 10.1164/ajrccm.158.supplement_2.13tac110 9817738

[10] . Emeryk-Maksymiuk J, Emeryk A, Krawczyk P, Wojas-Krawczyk K, Milanowski J. Beta-2-adrenoreceptor polymorphism at position 16 determines the clinical severity of chronic obstructive pulmonary disease. Pulm Pharmacol Ther. 2017;43:1-5. 10.1016/j.pupt.2017.01.005 28093224

[11] . Martinez FD, Graves PE, Baldini M, Solomon S, Erickson R. Association between genetic polymorphisms of the beta2-adrenoceptor and response to albuterol in children with and without a history of wheezing. J Clin Invest. 1997;100(12):3184-8. 10.1172/JCI119874 PMC508532 9399966

[12] . Akparova A, Aripova A, Abishev M, Kazhiyakhmetova B, Pirmanova A, Bersimbaev R. An investigation of the association between ADRB2 gene polymorphisms and asthma in Kazakh population. Clin Respir J. 2020; 14(6):514-20. 10.1111/crj.13160 32034992

[13] . Gupta S, Awasthi S. Pharmacogenomics of pediatric asthma. Indian J Hum Genet. 2010;16(3):111-8. 10.4103/0971-6866.73398 PMC3009420 21206697

[14] . McGraw DW, Forbes SL, Kramer LA, Liggett SB. Polymorphisms of the 5’ leader cistron of the human beta2-adrenergic receptor regulate receptor expression. J Clin Invest. 1998;102(11):1927-32. 10.1172/JCI4862 PMC509144 9835617

[15] . den Dunnen JT, Dalgleish R, Maglott DR, Hart RK, Greenblatt MS, McGowan-Jordan J, et al. HGVS recommendations for the description of sequence variants: 2016 Update. Hum Mutat. 2016;37(6):564-9. 10.1002/humu.22981 26931183

[16] . Tsai HJ, Shaikh N, Kho JY, Battle N, Naqvi M, Navarro D, Matallana H, Lilly CM, Eng CS, Kumar G, Thyne S, Watson HG, Meade K, LeNoir M, Choudhry S, Burchard EG; Study of African Americans, Asthma, Genes Environments (SAGE). Beta 2-adrenergic receptor polymorphisms: pharmacogenetic response to bronchodilator among African American asthmatics. Hum Genet. 2006;119(5):547-57. 10.1007/s00439-006-0169-2 16596417

[17] . Thomsen M, Nordestgaard BG, Sethi AA, Tybjærg-Hansen A, Dahl M. β2-adrenergic receptor polymorphisms, asthma and COPD: two large population-based studies. Eur Respir J. 2012;39(3):558-66. 10.1183/09031936.00023511 22075484

[18] . Carroll CL, Sala KA, Zucker AR, Schramm CM. Beta-adrenergic receptor polymorphisms associated with length of ICU stay in pediatric status asthmaticus. Pediatr Pulmonol. 2012;47(3):233-9. 10.1002/ppul.21544 21905268

[19] . Yoshida N, Nishimaki Y, Sugiyama M, Abe T, Tatsumi T, Tanoue A, et al. SNP genotyping in the beta(2)-adrenergic receptor by electronic microchip assay, DHPLC, and direct sequencing. J Hum Genet. 2002;47(9):500-3. 10.1007/s100380200074 12202992

[20] . Ducharme FM, Chalut D, Plotnick L, Savdie C, Kudirka D, Zhang X, et al. The Pediatric Respiratory Assessment Measure: a valid clinical score for assessing acute asthma severity from toddlers to teenagers. J Pediatr. 2008;152(4):476-80, 480.e1. 10.1016/j.jpeds.2007.08.034 18346499

[21] . U.S. National Library of Medicine. National Center for Biotechnology Information (NCBI). dbSNP. Bethesda: NCBI; s.d. [cited 2020 Dec 15]. Available from: https://www.ncbi.nlm.nih.gov/snp/

[22] . Mohamed-Hussein AA, Sayed SS, Eldien HM, Assar AM, Yehia FE. Beta 2 adrenergic receptor genetic polymorphisms in bronchial asthma: relationship to disease risk, severity, and treatment response. Lung. 2018;196(6):673-80. 10.1007/s00408-018-0153-3 30178312

[23] . Alghobashy AA, Elsharawy SA, Alkholy UM, Abdalmonem N, Abdou MA, Basset MA, et al. B_2_ adrenergic receptor gene polymorphism effect on childhood asthma severity and response to treatment. Pediatr Res. 2018;83(3):597-605. 10.1038/pr.2017.304 29658513

[24] . Ingebrigtsen TS, Vestbo J, Rode L, Marott JL, Lange P, Nordestgaard BG. β2-Adrenergic genotypes and risk of severe exacerbations in COPD: a prospective cohort study. Thorax. 2019;74(10):934-40. 10.1136/thoraxjnl-2018-212340 31481635

[25] . de Paiva AC, Marson FA, Ribeiro JD, Bertuzzo CS. Asthma: Gln27Glu and Arg16Gly polymorphisms of the beta2-adrenergic receptor gene as risk factors. Allergy Asthma Clin Immunol. 2014;10(1):8. 10.1186/1710-1492-10-8 PMC3930554 24499171

[26] . Scichilone N, Caponetto C, Fagone E, Benfante A, Paternò A, Heffler E, et al. The Arg/Arg polymorphism of the ADRB2 is associated with the severity of allergic asthma. J Allergy Clin Immunol Pract. 2016;4(6):1251-2. 10.1016/j.jaip.2016.06.014 27421901

[27] . Lipworth BJ, Basu K, Donald HP, Tavendale R, Macgregor DF, Ogston SA, et al. Tailored second-line therapy in asthmatic children with the Arg(16) genotype. Clin Sci (Lond). 2013;124(8):521-8. 10.1042/CS20120528 23126384

[28] . Sayers I. A tailored approach to asthma management: Arg(16) holds the key? Clin Sci (Lond). 2013;124(8):517-9. 10.1042/CS20120640 23205695

[29] . Turner S, Francis B, Vijverberg S, Pino-Yanes M, Maitland-van der Zee AH, Basu K, Bignell L, Mukhopadhyay S, Tavendale R, Palmer C, Hawcutt D, Pirmohamed M, Burchard EG, Lipworth B; Pharmacogenomics in Childhood Asthma Consortium. Childhood asthma exacerbations and the Arg16 β2-receptor polymorphism: a meta-analysis stratified by treatment. J Allergy Clin Immunol. 2016;138(1):107-13.e5. 10.1016/j.jaci.2015.10.045 PMC4931969 26774659

[30] . Rowe BH, Spooner C, Ducharme FM, Bretzlaff JA, Bota GW. Early emergency department treatment of acute asthma with systemic corticosteroids. Cochrane Database Syst Rev. 2001;(1):CD002178. Review. 10.1002/14651858.CD002178 11279756

[31] . Griffiths B, Kew KM. Intravenous magnesium sulfate for treating children with acute asthma in the emergency department. Cochrane Database Syst Rev. 2016;4(4):CD011050. Review. 10.1002/14651858.CD011050.pub2 PMC6599814 27126744

[32] . Craig SS, Dalziel SR, Powell CV, Graudins A, Babl FE, Lunny C. Interventions for escalation of therapy for acute exacerbations of asthma in children: an overview of Cochrane Reviews. Cochrane Database Syst Rev. 2020; 8:CD012977. 10.1002/14651858.CD012977.pub2 PMC8078579 32767571

[33] . Powell C, Kolamunnage-Dona R, Lowe J, Boland A, Petrou S, Doull I, Hood K, Williamson P; MAGNETIC study group. Magnesium sulphate in acute severe asthma in children (MAGNETIC): a randomised, placebo-controlled trial. Lancet Respir Med. 2013;1(4):301-8. Erratum in: Lancet Respir Med. 2013;1(4):285. 10.1016/S2213-2600(13)70037-7 24429155

[34] . Knightly R, Milan SJ, Hughes R, Knopp-Sihota JA, Rowe BH, Normansell R, et al. Inhaled magnesium sulfate in the treatment of acute asthma. Cochrane Database Syst Rev. 2017;11(11):CD003898. Review. 10.1002/14651858.CD003898.pub6 PMC6485984 29182799

[35] . Rodrigo GJ, Castro-Rodriguez JA. Heliox-driven β2-agonists nebulization for children and adults with acute asthma: a systematic review with meta-analysis. Ann Allergy Asthma Immunol. 2014;112(1):29-34. Review. 10.1016/j.anai.2013.09.024 24331390

